# The effect of health education on knowledge and behavior toward respiratory infectious diseases among students in Gansu, China: a quasi-natural experiment

**DOI:** 10.1186/s12889-020-08813-3

**Published:** 2020-05-13

**Authors:** Manli Wang, Haiqing Fang

**Affiliations:** 1 China Center for Special Economic Zone Research, Shenzhen University, Shenzhen, 518060 China; 2 Administration Office, Shenzhen People’s Hospital, Second Clinical Medical College of Jinan University, First Affiliated Hospital of Southern University of Science and Technology, Shenzhen, 518020 China

**Keywords:** Evaluation, Effect, Health education, Respiratory infectious diseases, Knowledge, Behavior, Students, Difference-in-differences analysis, China

## Abstract

**Background:**

The respiratory infectious diseases (RID) threaten the health and life quality of school students. However, previous related studies were insufficient in research design and method applied. This study aimed to evaluate the effect of health education on the knowledge and behavior of students toward RID through difference-in-difference (DID) analysis in Gansu, China.

**Methods:**

In 2015–2016, a one-year health education program in Gansu, China was conducted. The intervention group contained 1064 students before and 1001 students after the health education (2015 and 2016, respectively). The control group contained 1018 and 1001 students, respectively. The health education, including playing promotional cartoons, developing lectures, giving out handbook copies and making hand copy and blackboard newspapers, and publicity columns on RID, were conducted monthly from 2015 to 2016 in intervention group. The data were collected before and after the health education program with a questionnaire on the students’ knowledge and preventive behaviors regarding RID. The *×*^*2*^ and *t* tests were performed to compare the accuracy rate and scores for RID knowledge and behavior of the two groups. DID estimation was conducted to evaluate the effect of health education on RID knowledge and behavior while controlling the non- equilibrium variables.

**Results:**

After the health education program, the accuracy rate and scores of most items in the intervention group were significantly higher than those in the control group (*P < 0.05*) except for item k9 “What methods can prevent flu?”. The DID results wherein the demographics- age, nationality, and household register were controlled showed that health education significantly improved the accuracy rate of RID knowledge by 5.2–63.9% for most items, although the accuracy rates of items k2 “What’s the transmission way of the mumps?” and k9 were significantly decreased by 36.8 and 12.0%. The health education significantly improved the score of knowledge by 155.2% (*P < 0.001*) and the accuracy rate of all items of RID behavior by 2.9–51.5% except for item b3 “If you have phlegm, how do you usually deal with it?”. In addition, the health education also significantly improved the score of behavior toward RID of the sampled students by 138.2% (*P < 0.001*).

**Conclusion:**

The results of this study show that health education seemed to increase the RID knowledge and behavior of students. It is recommended that the health education should be enhanced and popularized in schools of China, and RID transmission routes and prevention methods should attract more attention.

## Background

Among the many types of respiratory infectious diseases (RID) [1] worldwide, mumps, tuberculosis (TB), and influenza are the most common [2, 3]. The Washington Post reported that approximately 150 cases of mumps have emerged and quickly infected more than 9000 people from January 2016 to June 2017 in America [4]. China also faces a fluctuating increasing trend of mumps, of which the incidence in 2013 was 24.20/100000 [5]. Mycobacteria tuberculosis [6] has infected approximately 2 billion people worldwide, accounting for one-third of the world’s population and includes 20 million active TB patients [7]. Influenza is another major risk factor harming human health and often leads to outbreaks or epidemics in different countries annually [8]. The emergence of these three types of RID endangers the physical and mental health of individuals and the safety of their life and property, threatens social stability, and imposes huge economic burden secondary to diseases worldwide, particularly to developing countries and region.

Among all groups of people worldwide, the primary, middle, and high school students form a special group. They are at the stage of body growth, characterized by tender immune function and low ability to resist RID [9–11], and their campus environment features high population density, close contact, and frequent RID communication, outbreaks and epidemics [12, 13]. In China, more than 70% of the public health emergencies occur in schools, and more than 80% of public health emergencies are infectious diseases epidemics [14, 15]. According to the 2014 National Internet-based Infectious Disease Reporting System, 4.02% of TB patients are students. The outbreak and epidemic of RID in schools threaten the health and quality of life of students. Moreover, such an event will disrupt the school’s teaching order, affect the happiness of the students’ families, and damage the stability and harmony of society [11, 16]. Therefore, considerable attention must be directed toward the RID issues among school students to prevent and control the spread of RID in school campuses.

Considering the current prevalence of RID in schools, most countries have implemented various measures for preventing and controlling the RID epidemic and outbreak to protect the health of school students [17]. Health education is an important strategy adopted by many schools in many countries. Health education is a planning, organizational, and systematic social education activity that enables individuals to consciously adopt healthy behavior and lifestyles to eliminate or mitigate the risk factors that affect health, prevent diseases, promote health, and improve the life quality of social activities [18]. Health education promotes the health awareness of students, improves their RID knowledge and directs their attention to RID prevention, thereby ameliorating their behavior toward RID. In 2017, the Health Ministry of China promulgated the “the 13th Five-Year Plan for National Health Promotion and Education” [19], which declared the necessity and urgency of carrying out health education to improve the health knowledge and behavior toward RID in counties and schools, including primary, middle, and high school students, and consequently improve their physical quality and health while preventing and controlling the spread of infectious diseases. For this endeavor, policymakers and researchers worldwide must identify the specific effect of implementing health education on people and students.

Some scholars have investigated the effect of health education on the prevention and control of RID of school students. In 2014, Xie et al. [20] found that the awareness for RID knowledge and the formation rate of healthy behavior of primary and high school students in the intervention group were significantly improved after the health education. In 2015, Yue et al. [21] examined the influence of face-to-face health education on the knowledge of mumps in primary school students and demonstrated that health education was suitable for the needs of primary school students to prevent and control of mumps and significantly improved their knowledge level on mumps. In 2016, Jee et al. [22] found that the scores of knowledge and attitudes of primary school students tended to improve after their exposure to TB prevention health education. Similarly, Juniarti et al. [23], Zhang et al. [24] and Mohammadi et al. [25] proved the health education could improve the knowledge, attitude, and behavior toward TB and other RID among adolescents and school students. All these studies confirmed the positive effect of health education on RID knowledge and behavior among school students [24, 26] by comparing the students’ RID knowledge and behavior before and after health education or between intervention and non-intervention groups. However, the improvement is affected by many factors, such as age, gender, educational level, and so on, the results and conclusions may not be completely attributed to health education program. Some other studies have used other scientific methods, such as difference-in-difference (DID) analysis, to analyze the effect of health education, [27] providing reference for health education evaluation more scientifically.

The health education toward RID prevention and control are highly valued by researchers and policy makers. Conducting evaluation of effect of health education on RID prevention among students has an important era, social and academic necessity. Located in a remote and bare area in northwestern China, Gansu Province [28] is relatively backward in economic and social conditions compared with other provinces and cities in eastern and central China and is in a relative shortage of education and health resources [28]. In 2015, a health education program toward RID among primary, middle and high school students was launched in QZ county in Gansu Province to improve the students’ RID prevention and control level. The program was carried out under the guidance of the China Health Education Center and the Gansu Provincial Health Administration. The health education was carried out through playing a cartoon painting, holding a RID knowledge lecture, issuing RID knowledge materials and producing texts, auditions, and languages in a variety of ways. We aimed to explore the knowledge and behavior changes of RID among the students and to explore the effect of health education through a DID estimation with taking Gansu as an example. The hypothesizes of this study are: 1) The RID knowledge and behavior of primary, middle and high school students will change before and after health education; 2) Health education has a good influence on RID knowledge and behavior of primary, middle and high school students.

Our study has some strengths. First, this was a quasi-natural experiment study, which was launched to improve the RID prevention and control level of the primary, middle, and high school students in two counties in Gansu Province. Some previous studies have evaluated the effects of health education on the RID prevention and control of students or adolescents in China and other countries. However, limited studies have explored the effect of RID health education through quasi-natural experiment. Our work filled this gap to a certain degree by implementing a health education program toward RID for primary, middle, and high school students. Second, this study aimed to evaluate the effect of the health education program on the knowledge and behavior of students toward RID through DID analysis in Gansu, China. DID was applied into the program evaluation by Ashenfelter and Card of Princeton University in 1985, and then it was introduced into the public health field by Yip W and Eggleston K in Harvard University and Conover C J in Duke University in 2001. Hereafter, DID was widely used in the medical field. DID can eliminate the objective effects through constructing intervention effects as key variables (double difference estimators) and controlling other covariates, to obtain an unbiased estimate of the results. Hence, compared with other relative study, the usage of DID made the results and conclusions more scientific and more reliable. Third, our study provided some strategies and reference for improving the health education program. At the same time, our research puts the health education of primary, middle and high school students on the agenda, which in turn helps the health administrative departments, schools and other institutions to strengthen the emphasis on the control of infectious diseases among school students.

## Methods

### Study design

This research is a quasi-natural experiment study. We intended to explore the effect of health education on knowledge and behavior toward RID among primary, middle and high school students in Gansu, China retrospectively. QZ county was regarded as the intervention group naturally, and WS was chosen as the control area. We assigned the 2015 unit as the pre-intervention period and 2016 as the post-intervention period, because of the hysteresis effects.

The study was divided into two parts. Firstly, we presented the accuracy rate and scores change of knowledge and behavior toward RID both in the intervention and control group before and after health education. Secondly, we explored the effect of health education on from DID estimation with the accuracy rate and scores of knowledge and behavior toward RID as the outcome variables, while adjusting for the non-equilibrium characteristics of the sampled students.

### Study settings

This research was concerned in Gansu province, northwestern China, which has a population of over 26 million in 2015 across a geographic area of 425,900 Km^2^. In 2015, Gansu province produced Gross Domestic Product (GDP) of 67.90 billion Ren Min Bi (RMB), ranking 29 among 34 provinces and special zones in China. At the same year, Gansu province’s GDP per capita was 26,600 RMB, which ranked the last in China. Gansu Province is relatively backward in social economy and faces a relative shortage of medical care and education resources.

**Fig. 1:**
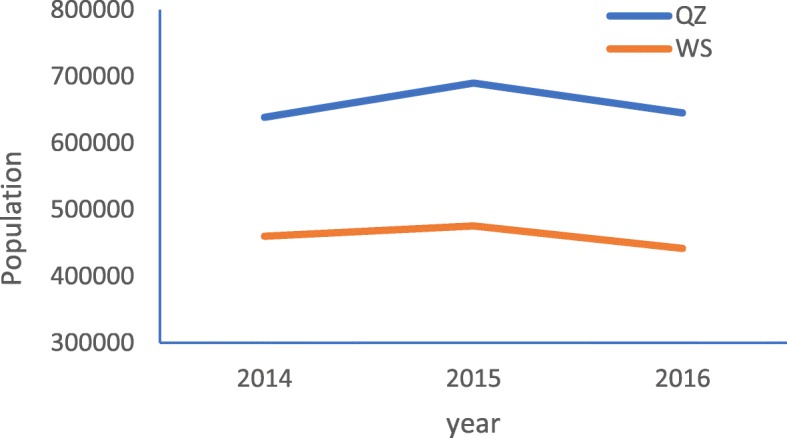
The population trend in QZ and WS county from 2014 to 2016

**Fig. 2:**
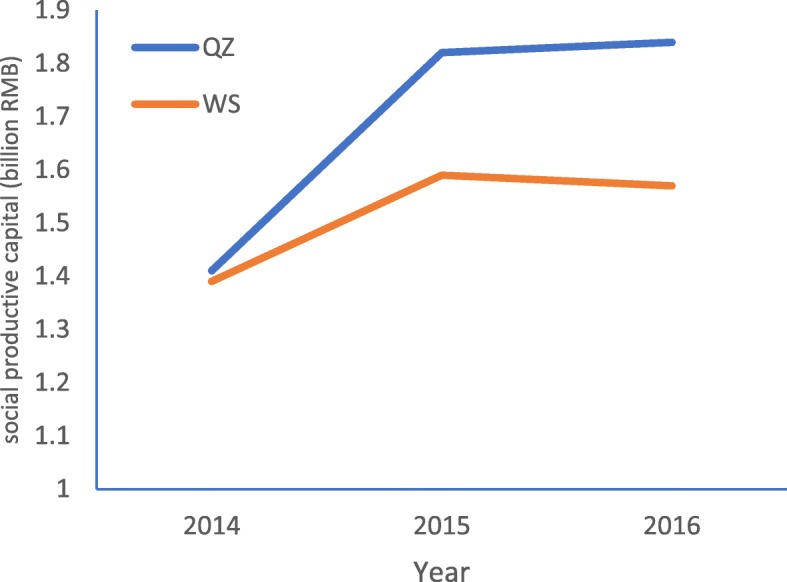
The trend of social productive capital in QZ and WS from 2014 to 2016

**Fig. 3:**
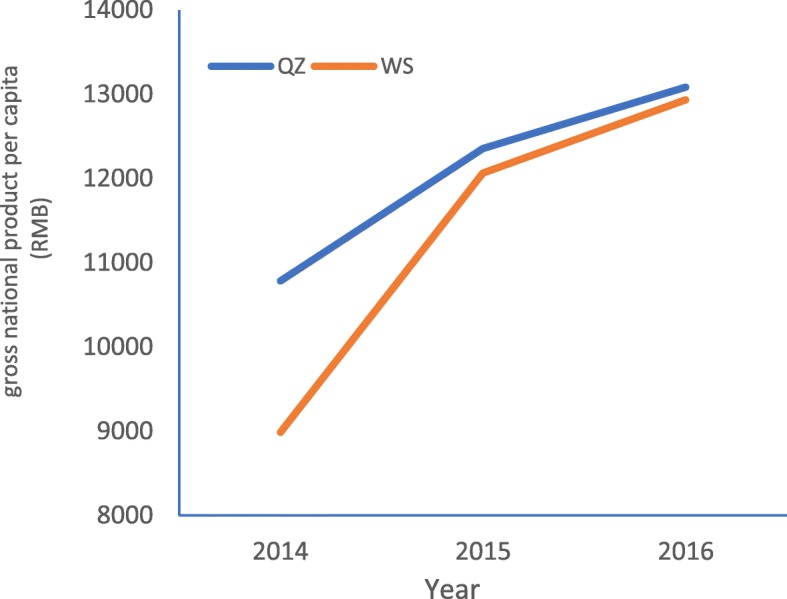
The trend of gross national product per capita in QZ and WS from 2014 to 2016

In order to improve the RID prevention and control level among students in some areas of Gansu Province, the Health Education Center of China began to implement a one-year health education program in QZ county of Gansu province in 2015–2016. Therefore, QZ county is naturally regarded as the intervention area. Then we selected WS county of Gansu province as the control area, which is near QZ county, to evaluate the effect of health education program with quasi-natural experiment. There are three reasons why we selected WS as the intervention area: 1) both QZ and WS are located in Southeastern Gansu province, and they have similar geographic location, natural conditions and education level; 2) not like QZ county, WS county didn’t receive the same or similar health education program during 2015 to 2016; 3) QZ and WS county were close to each other in terms of economic level, population size and geographical areage, and their development trend wete relatively consistent. In 2015, the geographical area of QZ and WS county was 2442 Km^2^ and 2011 Km^2^ respectively. Figures 1, 2 and 3 present the similar social and economic development trend of QZ and WS county from 2014 to 2016.

### Variables

Outcomes included the changes of the knowledge and behavior toward RID by the health education. Thus, the dependent variables included the accuracy rate and scores of knowledge and behavior toward RID, and the independent variables included the variable showing whether had the health education or not and the basic characteristic variables which included gender, age, nationality, education level, register area, and county of the sampled students.

For the characteristic variables, because gender, education level, and house register are categorical data, we directly grouped the categorical variables according to their original classification characteristics. For the age, we used the extreme value method to determine the maximum and minimum of it, and then changed the age into three categorical variables. This variable change was not only better to compare the difference between the intervention group and the control group on the age level, but also can maximally ensure the balance of the sample students, which was convenient for the next application of the DID method. For the nationality, there are 56 nationalities in China, of which the Han nationality has the largest number, and the other nationalities are usually collectively referred to as the minorities. Hence, in order to facilitate the comparative analysis, we classify all minority students into one category, making the nationality a binary variable.

The accuracy rate of knowledge and behavior toward RID, that is, the perception of students to choose the right answer to the knowledge and behavior items among all of the students within one group; For the accuracy rate, the formula is:

The accuracy rate of the RID knowledge = the number of respondents selecting the right answer to each RID knowledge item/the total number of respondents * 100%;

The accuracy rate of the RID behavior = the number of respondents selecting the right answer to each RID behavior item / the total number of respondents * 100%;

The scores of knowledge and behavior toward RID, that is, the total sum of the items that a responder answered correctly. For the scores, for each RID knowledge and behavior item, the responder received 1 score if he chose the right answer. The total sum of the nine knowledge items was 9, and the total sum of the six behavior items was 6. The formula of the mean value of RID knowledge/behavior scores is:

Mean value of RID knowledge scores = the scores’ sum of respondents answering to the RID knowledge /the total number of respondents;

Mean value of RID behavior scores = the scores’ sum of respondents answering to the RID behavior /the total number of respondents.

In addition to the overall knowledge and behavior scores of RID, we also examined the accuracy rate of each RID knowledge and behavior problem item. For RID prevention and control, every RID knowledge and behavioral problem item is quite important, and every item is the knowledge and behavior that students should master and be familiar with. These RID knowledge and behavior question items of RID, covering RID kinds, RID symptoms, transmission routes, prevention and control measures, treatment methods, etc., can examine the sample students’ perceptions on various aspects of RID in detail. Exploring the accuracy rate of RID knowledge and behavior items of the sample students not only can see the degree of mastery of each RID-related knowledge and behavioral skills of the sample students, but also can learn more about the effect of health education on various aspects of RID knowledge and behavior of the sample students. Understanding the status and changes in the RID knowledge and behavioral in detail will help to propose more targeted measures to improve students’ RID prevention level and improve health education programs.

### Overview of the health education program

In January 2015, the Health Education Center of China began to conduct monthly RID health education program on primary, middle and high school student in QZ county. The objective of this program was to improve the knowledge and behavior level toward RID of primary, middle, and high school students and consequently prevent and control the outbreak and epidemic of RID on campus effectively.

Before the health education program, a startup meeting was held, whose purpose was to inform the primary, middle and high school students and their teachers about the purpose, content, time of the health education and to encourage them to take part in the program positively. Before the implementation of the program, the main tasks also included contacting and training health education teachers and research teams, designing questionnaires, conducting pre-study, implementing questionnaire survey before health education, and preparing health education paper and electronic materials.

During this program, the health education was implemented for 12 times from 2015 to 2016, and the contents of the program were as follows: 1) playing promotional cartoons about RID awareness after gathering all the students from the intervention group into one meeting room; 2) developing RID lectures by the professional medical staff from the Center of Disease Prevention and Control in Gansu province; 3) giving out handbook copies on RID to every student in the intervention group; 4) making hand copy newspapers, blackboard newspapers, and publicity columns in the classes of intervention group. Taking into account the relative large group size, small age range (10–20 years old) and low education level of the sample students, the health education project adopted a collective form and developed through a combination of various methods including writing, language, audio-visual and so on, aim at maximizing the effect of the health education project and improving the RID knowledge and behavior level of the students.

After the health education program, the second meeting was carried out. This meeting was convened to thank participants for their cooperation, to collect and sort data and materials and assign the data analysis work.

### Study tool

The study tool used in this research was self-designed by the Health Education Institute in Gansu province. This self-designed questionnaire included the following contents: 1) the demographic information of the students- gender, age, nationality, educational level, and household register; 2) nine items concerning their knowledge on RID; and 3) six item relating to RID prevention behavior. Each item had various options to evaluate the sampled students’ responses.

Before the survey began, eight experts from public health fields such as tool construction, infectious disease prevention, and health education were invited to assess the content validity index of the questionnaire. The results showed that the content validity index (I-CVI) of the item level reached 0.81–1.00; among the content validity index (S-CVI) of the questionnaire level, the overall S-CVI (S-CVI /UA) was 0.86, and the average S-CVI (S-CVI /Ave) was 0.97. Related studies have shown that if the number of experts is greater than 6 people, the I-CVI is not less than 0.78, the S-CVI /UA is not less than 0.8 and the S-CVI /Ave reaches 0.90, then a good content validity of the questionnaire was proved [29]. Hence, the research tool used in this study have a good content validity.

In order to detect the structural validity of this questionnaire, we analyzed all the extracted data. After KMO and Bartlett spherical test, the KMO value was 0.78, the Bartlett spherical test had a *×*^*2*^ value of 7082.09 and a df value of 105 (*P < 0.001*), indicating that it was suitable for factor analysis. Exploratory factor analysis was performed using principal component analysis and maximum variance orthogonal rotation method, with the setting of extracting 2 common factors and deleting items with maximum factor load value < 0. Forty and items with double load. The exploratory factor analysis results did not delete any items, and the 9 knowledge items and the 6 behavioral items were divided into the different common factors. Each item has a load range of 0.41–0.78.

As for the reliability, the total Cronbach’s α coefficient of this questionnaire was 0.90, the Cronbach’s α coefficient of the knowledge dimension was 0.87, and the Cronbach’s α coefficient of the behavioral dimension was 0.88. At the same time, in the previous survey, the research team selected 100 people from the sample school, re-measured them after 2 weeks, and tested the test-retest reliability using the Pearson correlation coefficient. The test-retest reliability of the total questionnaire was 0.93, and the test-retest reliability of knowledge dimension and behavioral dimension was 0.92 (*P < 0.05*) and 0.95 (*P < 0.05*) respectively. This shows that the questionnaire used by the Institute had a good reliability.

### Data sources

The data in this study were derived from the original database of RID knowledge and behavior of residents, which was investigated, built and sorted by the Health Education Institute of Gansu province. In 2015 and 2016, under the help of Master and PhD students of School of Medicine and Health Management, Tongji Medical College, Huazhong University of Science and Technology, and School of Public Health in Lanzhou University, the Health Education Institute in Gansu province surveyed the health knowledge, behaviors and skills condition of residents through self-designed questionnaires.

We collected the data concerning primary, middle and high school students in QZ and WS county from the beginning of 2015 and mid-2016 as the data resources of this study through cluster sampling and systematic random sampling methods. The inclusion criteria for the sample students were: the primary, middle and high school students who had the willingness and ability to participate in the study and had no serious physical illness and mental illness; and the exclusion criteria for the sample students were: 1) Those who did not have the willingness and communication skills to participate in the study; 2) Students with severe physical or mental illness; 3) Primary, middle and high school students who are on vacation or are about to transfer. After screening the sample students according to the inclusion and dispatch criteria, for the data in 2015, QZ county had 1064 students (419, 313, and 332 in primary, middle, and high school, respectively); whereas WS county had 1018 participants (411, 303, and 304 in primary, middle and high school, respectively); for data in 2016, QZ county included 1001 students (419, 283, and 299 in primary, middle, and high school levels, respectively), and WS county included 1001 participants (384, 303, and 314, in primary, middle and high school, respectively). Because of getting sick or being not in school when the investigation began, students whose data are reported after the health education are not the same as those who were included in the sample before the health education.

### Statistical analysis

The STATA 12.0 (Stata Corporation, College Station, TX, USA) was used for statistical analysis. The statistical methods used in this work were conducted in three steps. 1) we conducted X^2^ test to examine the equilibrium of demographic characteristics (gender, age, nationality, educational level, and household register) between the intervention and control group samples. 2) the accuracy rate of knowledge and behavior toward RID and the scores of knowledge and behavior toward RID were compared between the intervention and control group samples through *X*^*2*^ and t test. 3) DID estimation was applied to assess the effects of the health education program on the accuracy rate and scores of knowledge and behavior toward RID while controlling for the non-equilibrium demographic characteristics of the sampled students in the framework of the regression model [30].

We considered the counterfactuals during estimation to validate our findings and reach a definitive conclusion, thus a difference-in-difference (DID) method was used to analyze the results. According to the research “Simplifying the estimation of difference in differences treatment effects with Stata” by Juan M. Villa from Munich Personal RePEc Archive, in the DID model, we defined g = 1 for the intervention group students, g = 0 for the control group students, t = 1 as after the health education program in 2016, and t = 0 as before the health education program in 2015. Let U_gt_ be the mean of an outcome variable in group g at time t. The difference in the means between the intervention and the control groups in 2016 (U_11_-U_01_) was the unadjusted estimate of the health education effect. By adjusting for the baseline difference, the adjusted estimate of the health education effect was expressed as (U_11_-U_01_) - (U_10_-U_00_), that is, the DID estimate of the health education effect. The DID estimator was the coefficient of the interaction term between intervention and time in a linear regression model where intervention, time, and their interaction served as covariates. For a binary outcome variable, the DID estimate was the difference between 2016 and 2015 in the proportion of students with the right option to one RID knowledge or behavior item. Moreover, the students’ non- equilibrium characteristics were regarded as covariates in the regression model.

In order to deal with the missing values of the data in this study, we read the relevant literature before the data analysis was carried out. According to the addressing method of missing values introduced in the book of “Medical Statistics (4th Edition)” by Sun Z and Xu Y, this study chose the mean substitution method to manage the missing data. It is relatively easy to replace the missing value with the mean value, and at the same time, it can keep the consistency of the data analysis results at a certain degree, which has some advantages.

### Quality control of this research

During the program design, data collection and data analysis stage, different measures have been taken to address the potential sources of bias and make quality control. ① During the program design stage, after multiple brainstorming sessions and literature analysis on health education evaluation, our research team chose QZ and WS in Gansu province of China as the intervention and control areas, who had similar economic and social development levels and relatively balanced population characteristics. The similarity of these two areas could avoid the impact of mixed effects on the evaluation, which may ensure the authenticity and scientific of the evaluation results. ② During the data collection stage, we screened the sample population in the database in strict accordance with individual exclusion and inclusion criteria. Then, we tested the reliability of the questionnaires to ensure the data quality. After calling in all the data, we checked and removed the unqualified data in time. ③ During the data analysis stage, in order to control the data bias, on the one hand, we adopted the DID analysis method to control the three imbalance variables- age, nationality and household register, thereby ensuring the evaluation results more scientific, reliable and close to the authenticity. On the other hand, we not only analyzed the accuracy rate of the single question items of RID knowledge and behavior, but also analyzed the scores of overall items. Such analysis can make the results more comprehensive and facilitate the discovery of more important conclusions.

## Results

### Demographic characteristics of the sampled students

Table 1 describes the demographic characteristics of the sampled students in the intervention and control groups. As shown in Table 1, the students in the two groups presented differences in their demographic characteristics, including age, nationality, and household register, indicating a non-equilibrium between the intervention and control groups.

**Table 1:** The basic characteristics of the intervention group and control group

Variable	Kinds	Intervention group (*n* = 2065)		Control group (*n* = 2019)		x2	*P*
		Counts	Frequency (%)	Counts	Frequency (%)		
Gender	Male	1131	54.77	1087	53.84	0.36	0.55
	Female	934	45.23	932	46.16		
Age	10–13 years old	955	46.25	857	42.45	7.07	0.03*
	14–17 years old	926	44.84	988	48.94		
	18–20 years old	184	8.91	174	8.62		
Nationality	Han	2024	98.02	1998	98.96	6.10	0.02*
	Minority	41	1.99	21	1.04		
Educational level	Primary school	838	40.58	795	39.38	1.96	0.38
	Junior high school	596	28.86	606	30.02		
	Senior high school	631	30.56	618	30.61		
Household register	Urban	1563	75.69	1168	57.85	146.65	< 0.01**
	Rural	502	24.31	851	42.15		

Inference: ** p < 0.01; * p < 0.05

The reason for the imbalance lies in the selection of the sample area, sample students and variable classification. This study took one county as the sample area for each group, the sample area is small, and the sample size is not large enough, and the sample classification leads to the statistics of the age, ethnicity and residence composition of these students. Learn the difference. In the subsequent DID analysis, we control these three variables to ensure that the results of the effect evaluation are not affected by its imbalance.

### Accuracy rate and scores of knowledge and behavior toward RID of the sampled students

Table 2 presents the accuracy rate and scores of knowledge and behavior toward RID both in the intervention and control group before and after health education.

**Table 2:** The accuracy and scores of knowledge and behavior before and after health education % (n)

Kinds	Items	Before health education		After health education		*P1*	*P2*	*P3*	*P4*	*Changes*	
		Intervention group (*n* = 1064)	Control group (*n* = 1018)	Intervention group (*n* = 1001)	Control group *(n* = 1001)					*Intervention group*	*Control group*
RID knowledge	k1. Which of the following are infectious diseases?	15.70 (167)	13.07 (133)	96.70 (968)	33.77 (338)	0.09	0.00^***^	0.00^***^	0.00^***^	81.01	83.64
	k2. What’s the transmission way of the mumps?	61.75 (657)	40.47 (412)	41.36 (414)	1.20 (12)	0.30	0.00^***^	0.00^***^	0.69	−20.39	0.89
	k3. What methods can prevent mumps?	21.52 (229)	16.60 (169)	97.30 (974)	28.87 (289)	0.00^**^	0.00^***^	0.00^***^	0.00^***^	75.78	80.70
	k4. What’s the transmission way of the TB?	76.60 (815)	69.65 (709)	99.40 (995)	80.42 (805)	0.00^***^	0.00^***^	0.00^***^	0.00^***^	22.80	29.76
	k5. What are the symptoms of TB?	82.43 (877)	74.95 (763)	99.90 (1000)	88.81 (889)	0.00^***^	0.00^***^	0.00^***^	0.00^***^	17.48	24.95
	k6. Whether the examination and treatment of TB is free in China?	9.96 (106)	80.94 (91)	38.26 (383)	13.79 (138)	0.45	0.00^***^	0.00^***^	0.00^**^	28.30	−42.68
	k7. Can the TB be cured?	71.81 (764)	62.28 (634)	96.00 (961)	74.93 (750)	0.00^***^	0.00^***^	0.00^***^	0.00^***^	24.20	33.73
	k8. What’s the transmission way of the flu?	56.67 (603)	57.27 (583)	94.51 (946)	52.15 (522)	0.79	0.00^***^	0.00^***^	0.02^*^	37.83	37.24
	k9. What methods can prevent flu?	22.37 (238)	16.01 (163)	16.78 (168)	11.99 (120)	0.00^***^	0.00^**^	0.00^***^	0.64	−5.59	−40.49
	Scores of knowledge towards infectious diseases (x̄±s)	3.95 ± 1.49	3.59 ± 1.56	6.35 ± 0.76	4.31 ± 1.53	0.21	0.000^***^	0.00^***^	0.42	–	–
RID behaviors	b1. Do you wash your hands before having a meal?	48.40 (515)	34.48 (351)	67.43 (675)	38.86 (389)	0.00^***^	0.00^***^	0.00^***^	0.04^*^	19.03	32.95
	b2. Do you wash your hands after you go to the toilet?	58.18 (619)	44.60 (454)	71.83 (719)	48.85 (489)	0.00^***^	0.00^***^	0.00^***^	0.06	13.65	27.23
	b3. If you have phlegm, how do you usually deal with it?	86.75 (923)	81.14 (826)	98.70 (988)	89.11 (892)	0.07	0.00^***^	0.00^***^	0.00^***^	11.95	17.56
	b4. Whether you cover your nose and mouth when coughing or sneezing?	48.40 (515)	38.41 (391)	71.33 (714)	47.75 (478)	0.00^***^	0.00^***^	0.00^***^	0.00^***^	22.93	32.92
	b5. If you found you have a fever in school, what should you do?	25.38 (270)	13.95 (142)	64.04 (641)	17.58 (176)	0.00^***^	0.00^***^	0.00^***^	0.03^*^	38.66	50.09
	b6. If you suspect yourself having TB, where will you go to see a doctor?	15.70 (167)	13.95 (142)	49.05 (491)	11.79 (118)	0.25	0.00^***^	0.00^***^	0.15	33.36	35.10
	Scores of behaviors towards infectious diseases (x̄±s)	2.85 ± 1.36	2.30 ± 1.37	4.223 ± 1.443	2.56 ± 1.33	0.45	0.02^*^	0.01^*^	0.62	–	–

*P1* was the *p*-values of the comparison of knowledge and behavior towards RID between the intervention and control groups before health education; P2 was the *p*-values of the comparison of knowledge and behavior towards RID between the intervention and control groups after health education; P3 was the *p*-values of the comparison of knowledge and behavior towards RID of intervention group before-after health education; P4 was the *p*-values of the comparison of knowledge and behavior towards RID of control group before-after health education

Inference: *** p < 0.001; ** p < 0.01; * p < 0.05

Before the health education program, among the 15 items (9 items of RID knowledge, and 6 items of RID behaviors), about 6 of the items’ accuracy rate comparison between these two groups had no statistical difference (*P > 0.05*), and the other 9 items had significant difference (*P < 0.05*). Before the health education program, the difference of RID knowledge scores (3.95 ± 1.49&3.59 ± 1.56, *P > 0.05*) and RID behavior scores (2.85 ± 1.36&2.30 ± 1.37, *P > 0.05*) between these the intervention and control groups had no statistical significance.

After the health education program, all the items’ accuracy rate concerning RID knowledge and behaviors in the intervention group was higher than those in the control group with significant difference (*P < 0.05*). Similarly, the intervention group students’ RID knowledge scores (6.35 ± 0.76&4.31 ± 1.53, *P < 0.05*) and RID behaviors scores (4.22 ± 1.44&2.56 ± 1.33, *P < 0.05*) were also significantly higher than those of the control group students.

Within the intervention group students, after receiving the health education, except for the item k9 “What methods can prevent flu?”, all the other RID knowledge and behavior items’ accuracy rate became significantly higher (*P* < 0.05). At the same time, the RID knowledge and behavior scores were also higher than those before the health education program with significant difference (*P* < 0.05). Before the health education, the accuracy rate of RID knowledge and behavior in the intervention group changed from 5.59 to 81.01%.

Within the control group, it had 5 RID knowledge and behavior items whose accuracy rate became lower, and only part of the items’ accuracy rate in the control group improved significantly than those before the health education. Simultaneously, within the control group, compared with those before the health education, although the scores of RID knowledge and behavior had increased, it had no statistical significance (*P* > 0.05). Before the health education, the accuracy rate of RID knowledge and behavior in the control group changed from 0.89 to 83.64%.

### Difference-in-difference estimation of the knowledge and behavior toward RID among sampled students

Table 3 describes the health education effects from DID estimation with the accuracy rate and scores of knowledge and behavior toward RID as the outcome variables adjusting for the non-equilibrium characteristics of the sampled students including age, nationality and household register. It is clear that the health education program does produce some impacts on the knowledge and behavior toward RID for the sample students in the intervention group.

**Table 3:** Health education effect on knowledge and behaviors towards infectious diseases: difference in difference (DID) estimation

Kinds	Items	No covariate				With covariates			
		DID estimator	SE	R^2^	*P*	DID estimator	SE	R^2^	*P*
knowledge towards infectious disease	k1. Which of the following are infectious diseases?	0.60	0.02	0.47	0.00***	0.62	0.02	0.49	0.00***
	k2. What’s the transmission way of the mumps?	−0.38	0.03	0.13	0.00***	−0.37	0.03	0.14	0.00***
	k3. What methods can prevent mumps?	0.64	0.02	0.44	0.00***	0.64	0.02	0.44	0.00***
	k4. What’s the transmission way of the TB?	0.12	0.02	0.08	0.00***	0.13	0.03	0.10	0.00***
	k5. What are the symptoms of TB?	0.04	0.02	0.07	0.08*	0.05	0.02	0.12	0.04**
	k6. Whether the examination and treatment of TB is free in China?	0.24	0.02	0.10	0.00***	0.05	0.02	0.32	0.01**
	k7. Can the TB be cured?	0.12	0.03	0.08	0.00***	0.12	0.03	0.09	0.00***
	k8. What’s the transmission way of the flu?	0.43	0.03	0.13	0.00***	0.43	0.03	0.14	0.00***
	k9. What methods can prevent flu?	−0.11	0.02	0.01	0.00***	−0.12	0.03	0.02	0.00***
	Scores of knowledge towards infectious diseases	1.68	0.09	0.37	0.00***	1.55	0.09	0.40	0.00***
behaviors towards infectious disease	b1. Do you wash your hands before having a meal?	0.15	0.03	0.06	0.00***	0.13	0.03	0.12	0.00***
	b2. Do you wash your hands after you go to the toilet?	0.09	0.03	0.04	0.00***	0.09	0.03	0.09	0.00***
	b3. If you have phlegm, how do you usually deal with it?	0.04	0.02	0.04	0.04**	0.03	0.02	0.08	0.15
	b4. Whether you cover your nose and mouth when coughing or sneezing?	0.14	0.03	0.05	0.00***	0.13	0.03	0.08	0.00***
	b5. If you found you have a fever in school, what should you do?	0.35	0.03	0.19	0.00***	0.49	0.03	0.26	0.00***
	b6. If you suspect yourself having TB, where will you go to see a doctor?	0.36	0.02	0.13	0.00***	0.52	0.02	0.24	0.00***
	Scores of behaviors towards infectious diseases	1.11	0.09	0.22	0.00***	1.38	0.09	0.33	0.00***

a. The Means and Standard Errors are estimated by linear regression; b. Inference: *** *p* < 0.01; ** *p* < 0.05; * *p* < 0.1. c. The R^2^, also known as the coefficient of determination, reflects the percentage of the dependent variables change which the linear model can explain. The range of R^2^ is 0–1, the larger the R^2^, the higher the interpretation degree of the dependent variables can explain the independent variables

In terms of the knowledge of RID, the health education had significantly improved the accuracy rate of RID knowledge by 5.20–63.9% for most of the items, although the accuracy rate of item k2 “What’s the transmission way of the mumps?” and item k9 “What methods can prevent flu?” had faced a significant decrease of 37.00 and 12.00%. The health education invention had improved the score of knowledge towards RID by 155.00% with statistical significance. For RID knowledge, the R^2^ value falls between 0.02–0.49, indicating that the health education can explain 2.00–49.00% of the change degree of accuracy rate or scores towards RID knowledge.

As for the RID behaviors, the health education had made the accuracy rate of all the items of RID behaviors improved by 3.00–52.00% with statistical significance except the item b3 “If you have phlegm, how do you usually deal with it?”. At the same time, the health education also had significantly improved the score of behaviors toward RID of the sample students by 138.00%. For RID behavior, the R^2^ value falls between 0.08–0.33, indicating that the health education can explain 8.00–33.00% of the change degree of accuracy rate or scores towards RID behavior.

## Discussion

The baseline survey results of this study showed that before the health education, both the accuracy rate of RID knowledge and behavior in the intervention and control groups were located in a low range. Compared with other provinces and cities in China, such as Beijing city [31] and Jiangsu Province [32], whose accuracy rate of RID among primary, middle, and high school students was much higher as showed in some relevant surveys, Gansu Province has a relatively lower awareness for most items of the knowledge and behavior toward RID, thereby showing the regional difference in RID awareness. Gansu Province is located in a remote and barren area in northwestern China, and its economic and social conditions are relatively poor [28]. In addition, its education resources are relatively scarce, which may responsible for the lack of opportunities for primary, middle, and high school students to receive health education in RID. Moreover, the residents, including students, of Gansu Province, China, are characterized by a generally low educational level, relatively weak infectious prevention awareness, and low health literacy [33], all of which may further reduce the accuracy rate and scores of infectious disease knowledge and prevention behavior among students.

Our results showed that compared with the control group, the intervention group presented significantly higher accuracy rate of most items of the RID knowledge and behavior and knowledge and behavior scores for primary, middle, and high school students after the health education intervention. The results of DID analysis confirmed the positive effect of health education on the increased accuracy rate and scores on RID knowledge and behavior. Our results are consistent with previous works that evaluated the effect of health education toward other infectious diseases, such as Wang et al. [34], Kang et al. [35], Al-Mazrou et al. [36], and Saleh et al. [37]. Our research is similar to other studies, mainly because health education program has similar positive effects on improving participants’ knowledge and behavioral towards various types of infectious diseases. This not only shows the importance of health education research regarding infectious diseases, but also shows that our research and findings are universal. In primary, middle, and high schools in China, students are busy with various exams and lack the awareness and enthusiasm for learning about RID. At the same time, Chinese schools often neglect teaching infectious disease-related courses, leading to a lack of health education toward RID among school students and hindering the improvement of the accuracy rate and scores of RID of these students. Such a phenomenon may be one of the main causes of the outbreak and epidemic of RID in primary, middle, and high school campuses. Our findings proved that health education for primary, middle, and high school students improved their awareness for preventing RID, improved their living and studying habits, and guided them in using the correct behavior to prevent and respond RID [38]. Furthermore, all of these measures can eliminate the spread of risk factors, protect the students from being infected by various RID, and decrease the incidence of RID in school campuses. Therefore, we believe that health education programs for RID should be promoted in primary, middle, and high schools to improve the awareness of these students about RID and optimize their prevention and control behavior toward RID.

In addition, our research found that although the health education program improved the accuracy rate of most RID knowledge and behavioral items, the accuracy rate of items “k2. What’s the transmission way of the mumps?” and k9 “What methods can prevent flu?” were significantly decreased by 36.80 and 12.00% under DID analysis. Moreover, the accuracy rate of item “b3. If you have phlegm, how do you usually deal with it?” was improved without significance. These items suggest that students do not know how to address RID when they actually face the RID and indicate the weaknesses and blind spots of the health education program. Correctly translating the RID knowledge into useful and actual prevention behavior is a difficult issue in the health education system [39]. On the one hand, the health education lacks teaching courses on behavioral imitation. On the other hand, the students’ forgetfulness of knowledge can also lead to ignorance and mistakes in prevention behavior. Furthermore, the lack of scientific assessment of the health education program, such as without DID method, prevents the comprehensive analysis of the insufficiency of health education. These problems may reduce the overall effect of infectious disease health education to a certain extent. Therefore, we believe that the health education program should deepen the knowledge on translating the infectious knowledge into correct prevention behavior among primary, middle, and high school students. At the same time, the detailed and scientific assessment of the effects of infectious disease health education must be enhanced and the shortcomings of the health education program must be actively and timely identified to guide the improvement of the health education system for students.

Our research confirmed the positive effects of health education on improving the accuracy rate and scores of RID among primary, middle, and high school students. In addition, our work identified the shortcomings of health education and proposed possible countermeasures for the RID knowledge items. The findings implied that the policymakers and health educators must continue working together in carrying out health education programs on campuses to improve the mastery of RID among primary, middle, and high school students. At the same time, the students must actively and cooperatively participate in the health education program actively and cooperatively. Moreover, to continue and deepen the implementation of health education, targeted health education activities should be conducted for primary, middle, and high school students to strengthen the education on weak knowledge and behavioral items and to optimize the curriculum of health education toward RID among primary, middle, and high schools. Furthermore, experts and scholars should enrich the assessment of infectious disease health education by using DID and other scientific methods to assess the effects of education and identify its shortcomings to improve the health education system for students.

Of course, our study also has some weaknesses. First, we selected only two counties in Gansu Province to implement the quasi-natural experiment of health education toward RID among primary, middle, and high school students. Such an approach may lead to the relatively low internal validity of this study. Therefore, when referring to the results of this study, the limitations of geography and samples should be considered. Second, conducting health education programs in schools may also affect the control group because of the unavoidable communication among students or teachers in different classes, which may pose certain risks to the stability of the results. For these weaknesses, we will continue to conduct and improve the research on health education of the students, so as to solve this study’s weaknesses and contribute to the improvement of the RID prevention and control for the primary, middle and high school students.

## Conclusion

The health education program positively affected the RID prevention knowledge and behavior of the sampled primary, middle, and high school students. For the control group students, a health education program should be applied to improve their knowledge and prevention behavior of RID. In addition, the implementation of the respiratory health education program should pay more attention to the weak items and parts of RID knowledge and behavior of primary, middle, and high school students. Moreover, the government, society, medical institutions, and schools should cooperate in promoting the popularization of infectious disease health education among students. Finally, the scientific and rational assessment of infectious disease health education should be strengthened to find the gaps of RID health education and consequently perfect the infectious health education system for school students.

## Data Availability

The datasets generated and analyzed for this study are not publicly available due to participant privacy but are available from the corresponding author upon reasonable request.

## Abbreviations

RID: Respiratory infectious diseases
DID: Difference in difference
TB: Tuberculosis
WHO: World Health Organization
GDP: Gross Domestic Product
RMB: Ren Min Bi
